# Guideline for the Diagnosis and Management of Heritable IFNAR1 Deficiency in Oceania

**DOI:** 10.1111/jpc.70421

**Published:** 2026-05-11

**Authors:** Cecilia Verryt, Paul Gray, Peter McNaughton, Jane Peake, Melanie Wong, George Aho, Emma Best, Maia Brewerton, Flora Lutui, Litara Esera Tulifau, Rebecca Qin, Satupa‘itea Viali, Petine White, Andrew Wood, See‐Tarn Woon, Theresa Cole, Alberto Pinzon Charry, Kuang‐Chih Hsiao

**Affiliations:** ^1^ Department of Clinical Immunology & Allergy Starship Children's Hospital Auckland New Zealand; ^2^ Department of Paediatrics: Child and Youth Health University of Auckland Auckland New Zealand; ^3^ Department of Paediatrics Tauranga Hospital Tauranga New Zealand; ^4^ Sydney Children's Hospital Randwick New South Wales Australia; ^5^ University of Western Sydney Sydney Australia; ^6^ Queensland Paediatric Immunology and Allergy Service Queensland Children's Hospital South Brisbane Queensland Australia; ^7^ University of Queensland St Lucia Queensland Australia; ^8^ The Children's Hospital at Westmead Westmead New South Wales Australia; ^9^ Paediatric Department Vaiola Hospital, Ministry of Health Nukualofa Tonga; ^10^ Department of Paediatric Infectious Diseases Starship Children's Hospital Auckland New Zealand; ^11^ Immunisation Advisory Centre The University of Auckland Auckland New Zealand; ^12^ Department of Clinical Immunology & Allergy Auckland City Hospital Auckland New Zealand; ^13^ Department of Microbiology and Immunology LabPLUS, Health New Zealand Auckland New Zealand; ^14^ Tupua Tamasese Meaole Hospital Apia Samoa; ^15^ Department of Allergy and Clinical Immunology Women's and Children's Hospital North Adelaide Australia; ^16^ Oceania University of Medicine Apia Samoa; ^17^ Department of Molecular Medicine and Pathology University of Auckland Auckland New Zealand; ^18^ Department of Oncology and Haematology Starship Children's Hospital Auckland New Zealand; ^19^ Department of Immunology and Allergy Royal Children's Hospital Parkville Victoria Australia; ^20^ Murdoch Children's Research Institute Parkville Victoria Australia; ^21^ Department of Paediatrics University of Melbourne Parkville Melbourne Australia; ^22^ Griffith University Nathan Queensland Australia

## Abstract

Autosomal recessive interferon alpha and beta receptor subunit 1 (IFNAR1) deficiency is a rare and heritable inborn error of immunity (IEI) predisposing individuals to severe and life‐threatening viral infections. It is more common in people of Western Polynesian ancestry, with estimates of around one in six thousand live births affected, due to being homozygous for or having two copies of the regionally relevant pathogenic *IFNAR1* variant c.1156G>T, p.Glu386*. IFNAR1 deficiency confers an increased risk of severe and life‐threatening infections caused by naturally circulating viruses including influenza, SARS‐CoV‐2, herpes simplex virus, respiratory syncytial virus (RSV), arboviruses and viruses in live attenuated vaccines (LAVs) including measles‐mumps‐rubella (MMR) and yellow fever. Complications including virus induced systemic hyperinflammation (VISH) are associated with significant mortality. This document outlines expert consensus regarding early identification, diagnostic workup and management of IFNAR1 deficiency in Australia, Aotearoa New Zealand and Western Pacific nations.

**Abbreviations**
HLH: haemophagocytic lymphohistiocytosis
IEI: inborn error of immunity
IFNAR1: interferon alpha and beta receptor subunit 1
IFNAR2: interferon alpha and beta receptor subunit 2
IFNg: interferon gamma
IFNα: interferon alpha
LAV: live attenuated virus
MMR: measles, mumps, rubella
NGS: next generation sequencing
PCR: polymerase chain reaction
VISH: virus induced systemic hyperinflammation
WES: whole exome sequencing
WGS: whole genome sequencing

## 1 Introduction

The Type I Interferon signalling pathway plays a pivotal role in innate antiviral defence. IFNα and β are induced following viral nucleic acid detection by innate immune receptors. The molecules then bind to the almost ubiquitously expressed Type I IFN receptor (IFNAR) composed of IFNAR1 and IFNAR2 subunits, effecting a proinflammatory antiviral response. There are a number of established heritable immunodeficiency syndromes associated with this pathway, such as deficiencies in STAT1, STAT2, IRF9, IRF7, MDA5. These have distinct but overlapping phenotypes, based on the specific genetic variant, completeness of penetrance, and variability in expressivity [1].

Deficiencies of the IFNAR subunits are recently described and predispose affected individuals to life‐threatening viral infections and hyperinflammation syndromes [2, 3]. IFNAR1 and IFNAR2 deficiencies are rare and together less than 50 individuals globally have been reported at the time of this publication [2, 4, 5, 6, 7, 8, 9, 10, 11, 12, 13, 14, 15, 16]. However, loss‐of‐function alleles do exist at higher frequencies in Western Polynesian (*IFNAR1* c.1156G>T, p.Glu386*) and Inuit (*IFNAR2* c.157 T>C, p.Ser53Pro) peoples (minor allele frequency of 0.0125 and 0.034 respectively) [2, 3, 13].

The clinical phenotype of autosomal recessive IFNAR1 and IFNAR2 deficiencies includes presentation in early childhood with severe and life‐threatening infection caused by a sentinel virus such as influenza, SARS‐CoV‐2, herpes simplex virus or measles [2, 3]. Of particular concern is susceptibility to viruses in LAVs including measles‐mumps‐rubella (MMR), the first dose of which is usually given between 12 and 18 months of age as part of the national immunisation schedules in Australia, Aotearoa New Zealand and many Pacific nations [2]. Notably, measles is a highly infectious disease with high morbidity and mortality in under‐vaccinated populations, brought into devastating focus during the 2019 measles outbreak in Samoa where the mortality rate for children under 5 years of age was 1 in 400 [17, 18, 19]. The contribution of IFNAR1 deficiency (homozygosity for c.1156G>T, p.Glu386*) to the overall mortality and morbidity of this outbreak is unknown but likely to be small, with an estimate of 1 in 6450 individuals affected by IFNAR1 deficiency [2]. Ensuring high vaccination coverage and maintaining effective community herd immunity is crucial for the health of all children.

This paper provides a review of the literature, expert consensus and available resourcing to provide clinical guidance in the Oceania region regarding when to test for IFNAR1 deficiency, and how to manage patients with suspected or confirmed IFNAR1 deficiency.

## 2 Red Flags for IFNAR1 Deficiency and Clinical Presentation

IFNAR1 deficiency can be difficult to recognise and many affected individuals appear healthy until they encounter their first infection caused by a sentinel virus. Early identification is crucial to prevent severe morbidity and mortality and relies on clinical case identification as there is currently no pre‐symptomatic screening beyond biological relatives of affected individuals. Based on expert consensus opinion and available literature, we recommend taking into account (1) a patient's ancestry, and (2) their personal and family history when considering the possible presence of autosomal recessive IFNAR1 deficiency syndrome due to homozygosity for *IFNAR1* c.1156G>T, p.Glu386* (Table 1).

**TABLE 1:** Decision‐making aid to support testing for IFNAR1 deficiency due to homozygosity for *IFNAR1* p.Glu386*.

Consider testing for IFNAR1 deficiency due to homozygosity for *IFNAR1* p.Glu386* in
A patient whose biological parent(s)^a^ report Western Polynesian and/or Māori ancestry.
AND a patient who:
Has or has had one or more life‐threatening viral infection(s)^b^ needing intensive care unit (ICU) or high dependency unit (HDU)‐level care or a fatal viral infection, OR b Has or has had two or more severe viral infections^c^ needing hospitalisation, OR c Has a family history of: ‐ an immediate family member (e.g., full siblings) having/having had severe, life‐threatening or fatal viral infections, or ‐ IFNAR1 deficiency due to homozygosity for (having two copies of) *IFNAR1* p.Glu386* variant

^a^ IFNAR1 deficiency is predominantly a recessively inherited condition. The *IFNAR1* p.Glu386* variant, which is more common in people of Western Polynesian ancestry, is a loss‐of‐function allele which results in IFNAR1 deficiency in individuals who are homozygous for this variant or compound heterozygous for this and another pathogenic variant.

^b^ Requiring paediatric intensive care admission, severe and/or multi‐organ involvement or invasive ventilation. Not adequately explained by another cause, for example, underlying medical condition, other known inborn error of immunity.

^c^ Requiring hospital admission with either multi‐organ involvement, central nervous system involvement, or respiratory involvement necessitating non‐invasive ventilation but not categorised as life‐threatening. Not adequately explained by another cause, for example, underlying medical condition, other known inborn error of immunity.

Firstly, autosomal recessive IFNAR1 deficiency caused by homozygosity for *IFNAR1* c.1156G>T, p.Glu386* is only possible when one pathogenic variant is inherited from each parent. This variant has been found with a minor allele frequency of 0.0125 in Samoa, and is also present in other countries in Western Polynesia [2]. While there is currently no publicly available information regarding this variant's frequency in Aotearoa Māori, one individual with both Māori and Western Polynesian ancestry has been reported to have IFNAR1 deficiency [2]. Within the current health record system in Aotearoa New Zealand, Māori is recorded as prioritised ethnicity, which may result in underlying Western Polynesian ancestry being less obvious to healthcare professionals relying on health record information. Therefore, the presence of either Western Polynesian and/or Māori ancestry is important and relevant when considering the possible presence of IFNAR1 deficiency.

To date, there are eight published cases of IFNAR1 deficiency due to homozygosity for *IFNAR1* c.1156G>T, pGlu386* from six kindreds, all of whom suffered their first severe viral illness before 16 months of age [2, 16]. Seven patients had illness attributed to LAV viral disease following MMR vaccination. Of these, six had confirmed acute systemic hyperinflammation, three died, while one suffered post‐infective sensorineural hearing loss and another exhibits global developmental delay. At the time of vaccine administration, three had no history of significant infections, two had a history of multiple short admissions with viral bronchiolitis, one had a prior episode of severe enteroviral meningitis and one had a sibling who suffered severe hyperinflammatory illness temporally related to MMR vaccination. One case tolerated two doses of MMR without complication, highlighting that previous tolerance of MMR does not completely rule out IFNAR1 deficiency [2]. Additionally, there is one previously unpublished case of IFNAR1 deficiency underlying fatal naturally acquired arbovirus‐associated systemic hyperinflammation syndrome [20]. During the 2014 chikungunya virus outbreak in Tonga, a 12‐month‐old boy born to non‐consanguineous parents was hospitalised with a 1 day history of fever, generalised maculopapular rash and pedal oedema and clinically diagnosed with chikungunya virus disease. Despite maximal medical therapy, he passed away from multi‐organ failure on Day 8 of infection. Post‐mortem genetic testing confirmed IFNAR1 deficiency due to homozygosity for *IFNAR1* p.Glu386* [20].

In comparison, among the 23 reported cases of other autosomal recessive IFNAR1 deficiency (due to homozygosity or compound heterozygosity for other recessively inherited loss‐of‐function variants) or autosomal recessive IFNAR2 deficiency which are phenotypically comparable to IFNAR1 deficiency, sixteen presented before 2 years of age, one of whom was diagnosed as a neonate due to known family history [3, 4, 5, 6, 7, 8, 9, 10, 11, 12, 13]. Fifteen cases document severe illness necessitating hospital admission attributed to vaccine strain viruses (MMR, Varicella, yellow fever) without prior sentinel illness; three patients have documented receipt of MMR vaccination with no ill effect [8, 11, 12]. Oral rotavirus, oral poliovirus, and BCG vaccines have not been observed to cause illness [8, 11]. In addition to LAVs, suspected or confirmed disease‐causing organisms associated with IFNAR1 and IFNAR2 deficiencies include influenza, SARS‐CoV‐2, RSV, and herpes simplex [2, 4, 6, 7, 8, 9, 13]. Concurrent polymerase chain reaction (PCR) detection of viruses including Epstein Barr Virus, Cytomegalovirus, Adenovirus, and Human Herpesvirus‐6 has been observed in a subset of patients in the setting of acute viral infection and complications but disease causality has not been established for all viruses [2, 6, 10, 13].

In summary, IFNAR1 deficiency should be considered in any individual of biparental Western Polynesian ancestry, presenting with personal or family history of severe or life‐threatening viral infections including systemic hyperinflammation syndrome.

## 3 Diagnosis of IFNAR1 Deficiency

Diagnostic work up for suspected IFNAR1 deficiency in Western Polynesian peoples can include functional and genetic testing. Diagnostic gold standard is detection of homozygosity for *IFNAR1* loss‐of‐function pathogenic variant c.1156G>T, p.Glu386* [2]. Table 2 outlines diagnostic methods available in Oceania. Test selection for an individual patient will depend on clinical index of suspicion, differential diagnoses, and resourcing.

**TABLE 2:** Comparison of diagnostic methods where *IFNAR1* p.Glu386* homozygosity is suspected.

Test	Availability	Advantages	Disadvantages	Cost	Timeframe
Clinical/Diagnostic laboratories					
Next generation sequencing (including whole exome or genome sequencing, gene panels)	Available through many accredited clinical/diagnostic laboratories worldwide (including, e.g., The Children's Hospital at Westmead, Sydney, Australia, Blueprint Genetics, Espoo, Finland)	Tests for a wide range of genetic conditions simultaneously. Provision of accurate clinical information will aid laboratory scientists in curating results.	Incomplete or inaccurate clinical information may result in genetic defect being missed or unreported. As knowledge is gained over time, previous negative results do not rule out novel disorders and require a request to re‐analyse.	High	Within 1–2 months
Hydrolysis probe genotyping (single nucleotide polymorphism detection)	Available through LabPLUS, Auckland (New Zealand)	Provides a cost‐effective, rapid and accurate result specific to IFNAR1 deficiency caused by p.Glu386*, permissive to using a range of sample types including dried blood spot	Will not identify other genetic variants in *IFNAR1* gene or in other genes in the interferon signalling pathway or associated with other inborn errors of immunity.	Low	Within a week
Sanger sequencing	Available through LabPLUS, Auckland and Canterbury Health Laboratories, Christchurch (New Zealand) and The Children's Hospital at Westmead, Westmead (Australia)	Identifies majority of variants in *IFNAR1* gene	Time and cost intensive. Will not identify genetic variants in other genes in the interferon signalling pathway or associated with other inborn errors of immunity.	Moderate	Within 1–2 weeks
STAT1 phosphorylation assay	Available through Royal Children's Hospital, Melbourne (Australia)	Provides direct evidence of functional defect in interferon signalling pathway	Will not identify specific genetic or autoimmune cause for the functional defect. Suitable only for local request. Blood to be tested within 24 h. Sending blood samples from outside of Australia is a logistic challenge.	Moderate	Within a week
Research laboratories					
Other research‐based methods in non‐accredited laboratories (including Western Blot for truncated protein, RNA/cDNA assay, flow cytometry for protein surface expression, immunofluorescence cell surface staining)	Universities and research laboratories	Characterisation of gene variant function and pathogenicity	Not widely available in accredited diagnostic laboratories IFNAR1 deficiency caused by p.Glu386* has already been functionally characterised.	Variable	Variable

If IFNAR1 deficiency is the only genetic condition under consideration, then limiting genetic testing to this gene reduces cost, result turnaround time and the potential for incidental findings. A novel genotyping assay designed to detect the c.1156G>T single nucleotide polymorphism (SNP) has been developed as a rapid and cost‐effective diagnostic tool for the common pathogenic IFNAR1 variant found in Oceania [21]. This method identifies the variant of relevance to our region; however, it will not identify other IFNAR1 variants or interferon pathway defects. The test is currently available through LabPLUS, Auckland, New Zealand. Local recommendations are to consider this test in a patient whose biological parents report Western Polynesian and/or Māori ancestry, and who, in addition, either have had a life‐threatening viral infection requiring high dependency care, or two or more severe viral infections needing hospitalisation, or an immediate family member with confirmed IFNAR1 deficiency or a suspicious clinical history.

Another approach to genetic diagnosis is Sanger sequencing of the full protein coding region of the IFNAR1 gene. This is more time‐ and cost‐intensive than a SNP‐detection genotyping assay. An example of where it might be beneficial is if the patient is heterozygous for p.Glu386* and manifesting severe viral infections, and there is concern that they may have a second different variant in the gene, although to date we have not yet encountered *IFNAR1* compound heterozygotes in Oceania.

If broader testing of IEIs is being considered, Next Generation Sequencing (NGS), including whole genome sequencing (WGS), whole exome sequencing (WES) and gene panels provides a broad range of genetic information and might be considered where there is a wider differential diagnosis [22]. Clinicians should be aware that results for WGS and WES are curated based on clinical information, and thus a prior negative result does not rule out more recently discovered genes or pathogenic variants. For example, the IFNAR1 gene is included in some but not all currently available Primary Immunodeficiency Gene Panels. Where the clinical picture has altered or time has passed, a re‐analysis may need to be requested.

Regarding functional testing, STAT1 phosphorylation assay can be used to assess the type 1 interferon signalling pathway. Reduced STAT1 phosphorylation identifies defective interferon pathway signalling; however, an abnormal result does not discriminate between primary and secondary causes. Moreover, many conditions affecting this pathway can yield similar results [1]. Other functional tests are available as research assays, which are typically used to establish pathogenicity of gene variants (Table 2).

In conclusion, if IFNAR1 deficiency is suspected in an individual reporting Western Polynesian and/or Māori ethnicities, our current advice would be to refer for an accurate, rapid and low‐cost genotyping test targeting the regionally relevant p.Glu386* variant. In the case of complex presentations or where the genotyping assay is negative, broader genetic testing may be considered.

## 4 Recommended Investigations and Management

Recommended investigations in patients suspected to have an IEI such as IFNAR1 deficiency that predisposes to viral infections are presented in Table 3.

**TABLE 3:** Investigations to consider in patients suspected to have viral immunodeficiency syndromes, due to family history, heritage, hyperinflammatory presentation or severe viral illness(es).

Investigations in patients suspected to have viral immunodeficiency syndromes*
*Investigations to consider*
Viral PCRs for viral pathogens as clinically indicated (e.g., based on exposure risk and history) on samples obtained from affected organ systems (including blood, CSF, bronchoalveolar lavage, tissue biopsies).
Blood‐based biomarkers: Full blood count* and film Electrolytes, renal and liver functions (including AST*, triglycerides*) Ig G/A/M Inflammatory biomarkers (CRP, ferritin*, ESR, LDH, sCD25*) Coagulopathy tests (fibrinogen*, D‐dimer, INR) Blood culture Lymphocyte subsets (including naïve and memory T cells; memory B cells, for children > 2 years) Vaccine antibody levels and viral serology (*These biomarkers contribute to the diagnostic criteria for systemic hyperinflammation conditions such as haemophagocytic lymphohistiocytosis and macrophage activation syndrome)
*Ancillary tests if clinically indicated*
Bone marrow +/− other tissue biopsy
Imaging of relevant organs
Investigation for underlying malignancies, rheumatological disease
Investigation for atypical infections (e.g., mosquito‐borne disease, mycobacteria, etc.)
*Diagnostic tests for IFNAR1 deficiency or other Primary Immunodeficiency*
Request single nucleotide polymorphism test where available
Where clinically indicated and accessible, request functional and/or genetic testing for primary (genetic) haemophagocytic lymphohistiocytosis (HLH) syndromes
Save DNA with family consent for later expansion of genetic testing if indicated

Patients with suspected IFNAR1 deficiency should undergo standard work‐up acutely to meet their clinical presentation. In addition, care should be taken to collect samples for evidence of inflammation and viral pathogen identification (including live attenuated strains) in relevant organ systems. This may include blood, cerebrospinal fluid, bronchoalveolar lavage and tissue biopsies. It is important to recognise the difference between results from serological tests and PCR tests. A positive serology test suggests that the patient's immune system has produced antibodies in response to exposure to the virus, but most serology tests cannot distinguish the source of exposure. A negative serology test does not rule out a recent or current infection, because antibodies may not have developed yet. Additionally, when interpreting serology results, clinicians should also consider any recent treatments, such as immunoglobulin or other immunomodulatory therapies, which may affect antibody levels. Detecting recent infection and viraemia requires PCR testing.

Biomarkers (Table 3) to assess for systemic hyperinflammation should be considered in any critically unwell child and assessed in the context of diagnostic criteria for hyperinflammatory syndromes [23]. If haemophagocytic lymphohistiocytosis (HLH), macrophage activation syndrome (MAS) or virus induced systemic hyperinflammation (VISH) syndrome is suspected, then a broad approach to investigating underlying aetiology should be applied [23].

DNA sample collection and genetic testing should be considered early. LAVs should be withheld during diagnostic work‐up. In the circumstance where LAVs are being withheld, the diagnostic process should be expedient and reasons communicated carefully to maintain confidence in routine immunisation programmes.

Table 4 outlines clinical management strategies and therapeutic interventions employed in IFNAR1 deficiency syndrome and appraises their strength using the NHMRC Levels of Evidence framework. Parenterally and intranasally administered viral LAVs such as MMR, yellow fever, live attenuated influenza (FluMist) vaccines are contraindicated. Caution is advised for viral LAV such as rotavirus and oral polio vaccines administered via the gastrointestinal tract because of limited data on effects in individuals with IFNAR1 deficiency. Patients should be proactively offered available non‐live or inactivated vaccines (e.g., COVID mRNA vaccine, inactivated Influenza), including consideration of off‐label subunit herpes zoster vaccine (Shingrix) for under 18 year olds. Vaccination of close contacts should be optimised. Passive immunisation should be considered. Consideration should be given to long‐term pooled immunoglobulin treatment, or if such option is not available, during periods of high community infection burden offer pre‐exposure prophylaxis (e.g., pooled immunoglobulin preparations, RSV antibody nirsevimab or palivizumab). All patients should have an emergency management plan. In acute illness, viral testing should be expedited, and prompt treatment initiated. Targeted viral treatments should be considered where a virus has been identified or strongly suspected (e.g., (val)acyclovir, oseltamivir, remdesivir). Overnight admission should be considered to help determine illness trajectory and enable early adjustments to treatment when indicated. Families should have access to diagnostic genetic tests and genetic counselling.

**TABLE 4:** Recommended management priorities in patients with confirmed IFNAR1 deficiency due to *IFNAR1* c.1156G>T, p.(Glu386*) homozygosity.

Recommended management in the community		
Withhold live viral vaccines containing measles, mumps, rubella, varicella, yellow fever, influenza (e.g., in FloMist), Japanese encephalitis (e.g., in Imojev) viruses	V	
Optimise active immunisation using non‐live vaccines including inactivated influenza, COVID (e.g., paediatric Comirnaty), human papillomavirus (HPV) and zoster recombinant subunit (e.g., Shingrix) vaccines	V	
Offer passive immunisation using pooled polyclonal antibody products (e.g., subcutaneous or intravenous immunoglobulin) long term, or if unavailable, monoclonal antibody products (e.g., palivizumab or nirsevimab for respiratory syncytial virus) when appropriate	IV	[2, 12, 16]
Evaluate adaptive immune response including vaccine antibody responses	V	
Prescribe post‐exposure prophylaxis (e.g., immunoglobulin for measles, zoster immunoglobulin for varicella, other appropriate polyclonal/monoclonal neutralising antibody preparations)	IV	[2]
Offer genetic counselling and family planning services including access to IVF, embryo selection and antenatal screening; testing of siblings	V	
Encourage pre‐conception, antenatal and postnatal optimisation of immunisation status (e.g., MMR, influenza, respiratory syncytial virus vaccines) of all household and close contacts	V	
Recommended management of acute presentations		
Offer rapid and comprehensive (broader range) viral PCR panel at first sign of acute viral symptoms	V	
Prescribe targeted antiviral medications (e.g., remdesivir for SARS‐CoV‐2, oseltamivir for influenza, (val)acyclovir for varicella and herpes simplex virus)	IV	[2, 4, 7, 12, 13]
Consider further investigations based on presentation (see Table 3)	V	
Consider overnight admission to determine illness trajectory if viral illness suspected or confirmed	V	
Consider in context		
Prescribe pre‐exposure prophylaxis, for example, palivizumab or nirsevimab for respiratory syncytial virus (RSV), other appropriate polyclonal/monoclonal neutralising antibody preparations in the setting of increased infection burden in the community	V	
Advise physical isolation measures in the setting of increased infection burden in the community	V	

### 4.1 Treatment of Acute Hyperinflammation Syndrome in IFNAR1 Deficiency

The treatment approach for acute hyperinflammation syndrome such as VISH syndrome in IFNAR1 deficiency remains complex and is characterised by a paucity of robust, high‐quality data. Therefore, clinical recommendations should be interpreted with caution and individualised based on clinical context and emerging evidence.

Rapid identification of any underlying viral infection and early implementation of targeted antiviral treatment are critical. Immunomodulatory doses of intravenous immunoglobulin have been used and appear to be well tolerated. Favourable clinical response has been observed in some patients with VISH [2]. The role of corticosteroids in VISH is not definitively established. While they are used in HLH and have been used to treat VISH, it remains unclear whether their net effect is beneficial or potentially harmful. IL‐1 receptor antagonists such as anakinra may offer benefit in some cases of secondary HLH. This is supported by limited data [24], but broader applicability and long‐term outcomes particularly in IFNAR1 deficiency remain uncertain. Emapalumab, a monoclonal antibody against interferon gamma (IFNg), has shown promise in treating various forms of HLH. However, in the context of IFNAR1 deficiency, IFNg may act as a critical compensatory mechanism during VISH. In such cases, inhibition of type II interferon pathway could be detrimental. Etoposide demonstrates relative specificity for activated T‐cells, while sparing naïve and memory T‐cells, macrophages, and dendritic cells [25] and has been used to treat pathogenic immune activation in HLH. However, its role in treating VISH in IFNAR1 deficiency by ablating activated T cells, some of which may have specificity against the viral trigger(s), remains questionable. Therefore, the role of emapalumab and etoposide remains unclear.

In summary, while these agents form part of the therapeutic interventions for HLH, their use in IFNAR1 deficiency and other deficiencies in type 1 interferon pathway must be guided by careful consideration of underlying pathophysiology, genetic context, and the limited evidence base. Immune suppression must be carefully balanced against the risk of viral persistence. On balance, we propose prioritising anti‐viral therapy alongside immunomodulatory doses of immunoglobulin and judicious use of steroids for IFNAR1‐deficient patients with VISH. Further research is urgently needed to clarify mechanisms driving VISH, optimise treatment strategies, and improve outcomes.

## 5 IFNAR1 Deficiency: Future Directions and Priorities

IFNAR1 deficiency is rare but of regional importance due to the increased prevalence in people of Western Polynesian ancestry, its association with heightened susceptibility to naturally acquired viral infections including SARS‐CoV‐2, influenza, herpes simplex, measles, and arboviruses, and links to adverse outcomes from a critical scheduled vaccine at a time when vaccination rates are ebbing. Equitable access across the wider Pacific region for tools to diagnose and treat IFNAR1 deficiency early is a key goal. This includes education of health professionals, diagnostic guidelines, access to investigations, and prompt treatment for acute illness in IFNAR1 deficiency.

A subset of infants will have sentinel viral infections prior to MMR and a growing number will have a family member with known IFNAR1 deficiency as more cases are diagnosed, which provides initial focus for targeted testing. Research and development of cost‐effective, accessible, and timely diagnostic methods is critical, and the recently developed and validated SNP‐detection genotyping assay is a candidate for upscaling in the region.

Pre‐symptomatic testing for IFNAR1 deficiency through inclusion in targeted or universal screening programmes may enable early diagnosis and early treatment. Detection in the newborn period is desirable as it offers the greatest opportunity to reduce the risk of life‐threatening viral infections through the earliest possible diagnosis. Such inclusion should be co‐designed with key stakeholders including indigenous health experts and community leaders, and deployed in a manner that further builds trust and confidence in public health and immunisation programmes. Maintaining high vaccine coverage and community herd immunity against measles is crucial especially as the region remains at very high risk of measles outbreaks which have devastating consequences at a population level [18, 19]. Immunocompromised individuals who are not recommended to have viral LAVs, such as those with IFNAR1 deficiency, are best protected through robust community herd immunity supported by high vaccination rates.

Development of novel non‐live vaccines against viruses such as measles may benefit vulnerable populations, including individuals with IFNAR1 deficiency and other patients who are immunocompromised. There is a collaborative effort led by researchers in Aotearoa New Zealand to develop a novel mRNA vaccine against measles to more equitably protect such populations.

## 6 Conclusion

IFNAR1 deficiency is a life‐threatening inborn error of immunity that is estimated to affect around one in six thousand individuals of Western Polynesian ancestry [2]. The clinical phenotype is narrow but includes heightened susceptibility to infections caused by naturally circulating viruses and viruses in LAVs. Early identification is crucial to reduce viral exposure and expedite management in case of infection in affected individuals. This can be achieved by increasing awareness among health professionals, provision of guidelines through the region for identification of suspected cases, diagnosis and management, and facilitation of cost‐effective, high throughput diagnostic tools.

## Funding

The authors have nothing to report.

## Conflicts of Interest

The authors declare no conflicts of interest.

## Data Availability

Data sharing not applicable to this article as no datasets were generated or analysed during the current study.
